# Recent Semen Exposure Impacts the Cytokine Response and Bacterial Vaginosis in Women

**DOI:** 10.3389/fimmu.2021.695201

**Published:** 2021-06-09

**Authors:** Khanyisile Mngomezulu, Gugulethu F. Mzobe, Andile Mtshali, Farzana Osman, Lenine J. P. Liebenberg, Nigel Garrett, Ravesh Singh, Anne Rompalo, Adrian Mindel, Salim S. Abdool Karim, Quarraisha Abdool Karim, Cheryl Baxter, Sinaye Ngcapu

**Affiliations:** ^1^ Centre for the AIDS Programme of Research in South Africa (CAPRISA), Nelson R Mandela School of Medicine, University of KwaZulu-Natal, Durban, South Africa; ^2^ Department of Medical Microbiology, Nelson R Mandela School of Medicine, University of KwaZulu-Natal, Durban, South Africa; ^3^ Department of Public Health, University of KwaZulu-Natal, Durban, South Africa; ^4^ Department of Microbiology, National Health Laboratory Services, KwaZulu-Natal Academic Complex, Inkosi Albert Luthuli Central Hospital, Durban, South Africa; ^5^ Department of Gynecology and Obstetrics, Johns Hopkins University, Baltimore, MD, United States; ^6^ Department of Epidemiology, Columbia University, New York, NY, United States

**Keywords:** semen exposure, prostate specific antigen, genital inflammation, cytokines, bacterial vaginosis

## Abstract

**Background:**

The presence of semen in the vagina from unprotected sex may influence the immune and microbial environment of the female genital tract. Inflammatory cytokine concentrations and BV-associated bacteria in female genital secretions may influence HIV risk, although the effect of recent sexual intercourse on incident BV and the cytokine milieu of cervicovaginal secretions has rarely been measured in previous studies. Here, we investigated the extent to which partner semen impacts the cytokine response and incident BV.

**Methods:**

At baseline, we assessed the recency of semen exposure in menstrual cup supernatants by quantifying prostate specific antigen (PSA) levels using ELISA in 248 HIV-uninfected women at high risk for HIV infection. Luminex was used to measure 48 cytokines in menstrual cup supernatants and vaginal swabs to diagnose BV by Nugent score. Point-of-care screening for *Chlamydia trachomatis* and *Neisseria gonorrhoeae* was conducted using GeneXpert while OSOM was used for *Trichomonas vaginalis* detection. Multivariable models, adjusted for age, sexually transmitted infections, BV, current contraception use and condom use, were used to assess the impact of semen exposure on biomarkers of inflammation and BV.

**Results:**

Presence of PSA, indicating recent semen exposure within 48 hours prior to sampling, was observed in menstrual cup supernatants of 17% (43/248) of women. Of these women, 70% (30/43) had self-reported condom use at their last sex act and 84% (36/43) had BV (Nugent score >7). PSA presence was significantly associated with prevalent BV (Relative Risk (RR), 2.609; 95% Confidence Interval (CI), 1.104 - 6.165; p = 0.029). Furthermore, women with detectable PSA had high median concentrations of macrophage inflammatory protein- beta (MIP-1α, p=0.047) and low median concentration of the stem cell growth factor beta (SCGF-β, p=0.038) compared to those without PSA.

**Conclusion:**

A degree of discordance between self-reports of consistent condom use and PSA positivity was observed. There was also evidence of a relationship between recent semen exposure, BV prevalence and altered cytokine concentrations. These findings suggest that PSA, as a semen biomarker, should be taken into consideration when investigating biological markers in the female genital tract and self-reported condom use in studies on reproductive and sexual health.

## Introduction

Semen exposure through unprotected sexual intercourse as well as inconsistent and incorrect condom use may result in heterosexual transmission of HIV, likely by modulating the inflammatory response and altering optimal vaginal microbial communities of the female genital tract (FGT) (1–4). Both high levels of inflammatory cytokines and diverse microbial communities in the FGT have been associated with elevated genital inflammation and increased HIV risk (5–7). Furthermore, semen may impact both physiological and patho-physiological events in the FGT. This includes tissue remodelling, response to foreign antigens in seminal fluid and bacterial and viral infections such as HIV (2).

Several studies have demonstrated that the presence of semen in the vagina during unprotected sex is associated with an inflammatory response and short-term activation of mucosal immunity (8–10). In addition to spermatozoa, seminal fluid contains alkaline pH, potent anti-inflammatory cytokines (Transforming growth factor-beta (TGF-β), Interleukin (IL)-10, Prostaglandin E2 (PGE2), and pro-inflammatory cytokines [IL-8, secretory leukocyte protease (SLP)-1], all with the capacity to alter the immune environment of the vaginal mucosa (8, 11, 12). Seminal fluid also contains signalling molecules that increased expression of IL-1 beta (IL-1β), IL-6 and leukaemia inhibitory factor (LIF) by endometrial epithelial cells *in vitro* (1, 11, 13). Furthermore, *in vitro* studies of the endometrial epithelial cells showed that human seminal plasma decreased the secretions of innate antiviral factors (e.g. secretory leukocyte protease inhibitor), while increasing a cascade of inflammatory cytokines and chemokines (Granulocyte macrophage colony-stimulating factor (GM-CSF), IL-1 alpha (IL-1α), IL-1β, Growth regulating alpha (GROα), Macrophage inflammatory protein- alpha [MIP-1α), MIP-1β, MIP-3 alpha (MIP-3α)] as well as the chemokine ligand for CC chemokine receptor 6 (CCR6) receptor expressed by cluster of differentiation 4 (CD4+) T helper (Th)-17 cells and Langerhans cells (8, 11, 12, 14). Expression of these cytokines is known to trigger the recruitment and activation of susceptible cells (11), suggesting that semen can increase a woman’s susceptibility to HIV, or other sexually transmitted infections (STIs).

Another contributing factor responsible for semen-induced immunity and inflammatory responses is the presence of microbial communities within semen, which have the potential to alter the composition of the vaginal microbiota (15–17). After unprotected sexual intercourse, the seminal microbial communities have been associated with a significant decrease in the relative abundance of the naturally occurring *Lactobacillus* species and an increased relative abundance of diverse bacterial species linked to bacterial vaginosis (BV) (15–18). Recently, studies examining the vaginal microbiota by sequencing the 16S rRNA bacterial gene showed diverse vaginal microbiota in young women with elevated inflammation, which subsequently led to increased HIV risk by inducing the mucosal HIV target cell frequency and activation (19, 20). The diversity of microbial assemblages have also been shown to increase HIV risk by weakening the mucosal epithelial barrier function and reducing protective factors such as antimicrobial agents (21).

Given the impact that unprotected sexual intercourse has on the vaginal immune response and microbiome, an objective assessment of semen exposure is needed to accurately interpret mucosal immunity and BV data from vaginal fluids of women enrolled in HIV prevention trials. Measurement of semen exposure using biomarkers has been identified as a robust method to reduce the reliance on self-reported sexual activity in studies investigating immunological factors in the FGT, risk of infection or probability of pregnancy (22–26). Two semen biomarkers; prostate-specific antigen (PSA) and the Y-chromosome DNA, have been used to indicate the presence of semen within the FGT (27, 28). Y-chromosome DNA is detectable for up to 2 weeks post sexual intercourse, using a polymerase chain reaction (PCR) based assay (29, 30), while the PSA protein has a short half-life of 48 hours within the vaginal tract. The PSA, which can be found in high concentrations in vaginal fluids obtained from self-collected swabs following recent semen exposure (31–33), is more frequently used as a surrogate indicator for unprotected sexual intercourse than the Y-chromosome DNA (3, 34, 35). However, very few studies have used PSA to control for the potential confounding effect of semen in the FGT (3, 36). Most studies use self-reported frequency of sex, the number of partners and condom use to control for confounding (19, 37–39).

There is a need to better understand the concordance between self-reported consistent condom use and the presence of semen biomarkers, as measured by PSA. Understanding the impact of semen exposure on FGT cytokine milieu and microbial communities is also important. The purpose of this study was to assess the concordance between self-reported consistent condom use and the presence of semen biomarker; to evaluate the extent to which partner semen alter cytokine profiles; and to investigate the relationship between semen exposure and incident STIs and BV.

## Methods and Materials

### Study Design, Participants and Specimen Collection

Cervicovaginal samples were collected from 248 women undergoing STI management in the CAPRISA 083 study. CAPRISA 083 was a prospective study aimed at reducing STIs in women by enhancing the STI management package offered for targeted laboratory diagnosed STI care, ensuring that the individual is cured and reducing the risk of reinfection using expedited partner therapy (40). Participant demographics and clinical data were collected at enrolment using a structured questionnaire. At enrolment, vulvovaginal swabs were collected from the posterior fornices and lateral vaginal walls, followed by inserting a menstrual cup (SoftCup^®^, EuroFemPro, Netherlands) for one hour to collect genital secretions for both microbial and immunological assays. Study participants were eligible for enrolment if they were female, aged 18-40 years and HIV-1 uninfected. Participants were not eligible for enrolment if they were menstruating at sample collection, pregnant, disclosed any form of sex work, and women who have had any antibiotic treatment within the last 7 days. Point-of-care STI screening was performed using GeneXpert (Cepheid, North America) assays for *Chlamydia trachomatis and Neisseria gonorrhoea.* OSOM rapid test was used to test for *Trichomonas vaginalis*. Assessment for these STIs was conducted using the wet prep and results were confirmed with PCR. Women infected with *C. trachomatis* were treated with 1g azithromycin orally and *N. gonorrhoea* with 250mg ceftriaxone intramuscularly. BV was determined by Nugent score (score of < 3 was regarded as normal vaginal flora, 4-6 as intermediate flora and 7-10 as BV). Women who were diagnosed with intermediate flora, BV and *T. vaginalis* were offered a single dose of oral metronidazole 2g.

### ELISA to Detect Prostate Specific Antigen (PSA)

Human tissue kallikrein 3 (R&D Systems, Quantikine ELISA, Inc., Minneapolis, USA), commonly known as PSA, was measured in menstrual cup supernatants using ELISA assay. Briefly, 50 µl of menstrual cup supernatant was used for PSA detection, with upper limit of detection of 60 ng/ml and a threshold positivity of 0.94 ng/ml, as per manufacturer’s protocol. Every plate included PSA standards (provided in the kit) and negative control containing sterile PCR-grade water and reaction mix. The average absorbance values for each set of reference standards, negative control, positive control and the samples were measured at 450 nm wavelength using the VersaMax™ absorbance microplate reader (Molecular Devices, Inc., Sunnyvale, USA).

### Cytokine Measurements

At baseline, concentration levels of 48 cytokines were detected in menstrual cup supernatants and expressed in log_10_ (pg/ml). The cytokine panel included chemokines, pro-inflammatory cytokines, adaptive, growth factors and anti-inflammatory: Interleukin (IL)-1β, IL-1Rα, IL-2, IL-4, IL-5, IL-6, IL-7, IL-8, IL-9, IL-10, IL-12p70, IL-12p40, IL-16, IL-18, IL-1A, IL-2RA, IL-3, IL-13, IL-15, IL-17, basic fibroblast growth factor (FGF-basic), cutaneous T-cell attracting chemokine (CTACK), Eotaxin, granulocyte colony-stimulating factor (G-CSF), GM–CSF,GRO-α, hepatocyte growth factor (HGF), Interferon (IFN)-γ, IFN-α2, interferon-γ -inducible protein (IP)-10, leukaemia inhibitory factor (LIF), monocyte chemoattractant protein (MCP)-1, MCP-3, macrophage colony-stimulating factor (M-CSF), monokine induced by gamma- interferon (MIG), Macrophage migration inhibitory factor (MIF), macrophage inhibitory protein (MIP)–1α, MIP-1β, nerve growth factor-beta (NGF-β), platelet derived growth factor (PDGF-ββ), regulated upon activation normal T cell expressed and presumably secreted (RANTES),stem cell factor (SCF), stem cell growth factor-beta (SCGF-β), stromal cell-derived factors 1- alpha (SDF-1α), tumour necrosis factor alpha (TNF)–α, TNF-β, TNF-related apoptosis-inducing ligand (TRAIL), and vascular endothelial growth factor (VEGF) were measured using the Bio-Plex Pro Human Cytokine kits Group I (27-Plex Panel) and Group II (21-Plex Panel) in a Bio-Plex Reader™200 system (Bio-Rad Laboratories, USA). Assays were performed according to the manufacturer’s protocol. Menstrual cup supernatants were thawed overnight on ice and filtered by centrifugation using 0.2 μm cellulose acetate filters (Sigma, USA). Bio-Plex manager software (version 5.0; Bio-Rad Laboratories Inc^®^, USA) was also used to analyse the data and all analyte concentrations were extrapolated from the standard curves using a 5 PL regression equation. Analyte concentrations that were below the lower limit of detection of the assay were reported as the mid-point between zero and the lowest concentration measured for each analyte.

### Statistical Analysis

Descriptive statistics were summarized using medians and interquartile ranges for continuous variables and proportions for categorical variables. The Fisher’s exact test was used to compare proportions between groups, whilst the Wilcoxon rank sums test was used to compare two medians. To measure the impact of semen exposure on cytokine concentrations, linear regression models were fitted to log-transformed cytokine concentrations. Multivariable models were adjusted for age, STI (*C. trachomatis*, *N. gonorrhoea and T. vaginalis*), BV, current contraception use and condom use. Statistical analyses were conducted using GraphPad Prism 7.05 (GraphPad Software, USA) and SAS version 9.4 (SAS Institute Inc., Cary).

## Results

### Clinical and Socio-Behavioural Characteristics of the Study Participants

This study included 248 women with the median age being 23 years (interquartile range (IQR) 21-27 years). Of these, 17% (43/248) tested positive for PSA and 83% (205/248) had no detectable PSA in the menstrual cup supernatants by ELISA. About 70% (30/43) of the women who reported any condom use with their partner to prevent STIs tested positive for PSA, suggesting that condom use was likely over-reported or they engaged in unprotected sexual intercourse 48 hours before sample collection. PSA was detected in 20% (13/66) of menstrual cup supernatants from women who reported no condom use with a partner. Although not statistically significant, PSA was more frequently detected in women using progesterone based injectables 83% (10/12) compared to other forms of contraceptive users [oral-contraceptive pill 8.3% (1/12), subdermal implant 8.3% (1/12) and condoms (0)].

### Prevalence of BV and STIs in PSA Positive *Versus* PSA Negative Women

In **Table 1**, we also examined the relationship between PSA and prevalent BV or STIs. Of the 248 women who were screened for BV, 31% (76/248) had a normal vaginal flora as indicated by Nugent score of < 3 (dominated by *Lactobacillus* spp.), 35% (87/248) had intermediate BV (Nugent score 4-6, with a diversity of bacteria) and 34% (85/248) had BV (Nugent score >7, with a diversity of anaerobic bacteria). BV prevalence did not differ amongst women whom PSA was detected (34.9%, 15/43) had compared PSA-negative women 34.1% (70/205) (**Table 1**). At baseline, 14% (35/248) of women were infected with *C. trachomatis*, followed by *N. gonorrhoeae* (4%, 11/248) and *T. vaginalis* (4%, 9/248). PSA was detected in 23% (8/35) of women with *C. trachomatis*, 11% (1/9) in those with *N. gonorrhoeae* and 9% (1/11) in those with *T. vaginalis*.

**Table 1:** Baseline participant demographics according to presence of PSA in genital secretions.

Variable	Level	Overall	PSA+	PSA-	P value
		N=248	N=43	N=205	
		% (n/N) or Median (IQR)			
**Age (Years)**	Median (IQR)	23 (21 - 27)	23 (21 - 27)	23 (21- 26)	0.291
**Highest level of education**	Primary	0.4 (1/244)	0	0.5 (1/201)	0.651
	Secondary	72.5 (177/244)	76.7 (33)	71.6 (144/201)	
	Tertiary	27.0 (66/244)	23.3 (10)	27.9 (56/201)	
**Condoms use with partner(s) to protect yourself from STIs**	Yes	73.4 (182)	69.8 (30)	74.1 (152)	0.572
	No	26.6 (66)	30.2 (13)	25.9 (53)	
**Frequency of condom use**	Always	4.0 (10)	0	4.9 (10)	0.290
	Sometimes	64.9 (161)	62.8 (27)	65.4 (134)	
	Never	31.0 (77)	37.2 (16)	29.8 (61)	
**Contraception use or practicing any form of birth control**	Yes	35.9 (89)	27.9 (12)	37.6 (77)	0.294
	No	64.1 (159)	72.1 (31)	62.4 (128)	
**Contraception type**	Condom only	7.9 (7/89)	0	9.1 (7/77)	0.590
	Oral-contraceptive pill	11.2 (10/89)	8.3 (1/12)	11.7 (9/77)	
	Progesterone injections	58.4 (52/89)	83.3 (10/12)	54.5 (42/77)	
	Subdermal Implant	20.2 (18/89)	8.3 (1/12)	22.1 (17/77)	
	Intra-uterine device (IUD)	2.2 (2/89)	0	2.6 (2/77)	
**Bacterial vaginosis status**	Normal	30.6 (76)	16.3 (7)	33.7 (69)	0.038*
	Intermediate	35.1 (87)	48.8 (21)	32.2 (66)	
	BV	34.3 (85)	34.9 (15)	34.1 (70)
**Sexual transmitted infections**					
*T. vaginalis*	Positive	3.6 (9)	2.3 (1)	3.9 (8)	1.000
	Negative	96.4 (239)	97.7 (42)	96.1 (197)	
*C. trachomatis*	Positive	14.1 (35)	18.6 (8)	13.2 (27)	0.342
	Negative	85.9 (213)	81.4 (35)	86.8 (178)	
*N. gonorrhoeae*	Positive	4.4 (11)	2.3 (1)	4.9 (10)	0.695
	Negative	95.6 (237)	97.7 (42)	95.1 (195)	
**Any STI (*C. trachomatis*, *N. gonorrhoeae* or *T. vaginalis*)**	Positive	20.2 (50)	23.3 (10)	19.5 (40)	0.539
	Negative	79.8 (198)	76.7 (33)	80.5 (165)	

*P < 0.05, PSA, prostate specific antigen; C, trachomatis-Chlamydia Trachomatis; N, gonorrhoeae -Neisseria gonorrhoeae; T, vaginalis-Trichomonas vaginalis; BV, bacterial vaginosis; STIs, sexually transmitted infections; IQR, interquartile range. Descriptive statistics are reported as medians and IQRs (continuous data) or percentages (categorical data). Numbers were not the same in some groups due to missing data. PSA concentrations greater than 1.0 ng/mL were considered as providing evidence of semen exposure within the past 2 days. Values shown in bold were those that were significant p < 0.05.

We then assessed the relative risk of acquiring BV or STIs in women in whom PSA was detected using a logistic regression model. After adjusting for potential confounders (age, current contraceptive use and condom use), PSA was significantly associated with prevalent BV based on clinical symptoms and Nugent score >4 (aRR, 2.607; 95% CI, 1.086 - 6.258; p=0.032) (**Table 2**). In contrast, we observed no significant association between recent unprotected sex, as measured by PSA, and relative risk of acquiring STIs (RR, 1.074; 95% CI, 2.419 – 0.476; p = 0.864) (**Table 2**).

**Table 2:** Associations between PSA positivity, STI and BV.

Characteristics	Level	Relative Risk	Standard Error	95% Confidence Interval		P value
				Lower	Upper	
**PSA vs BV**	Negative	Ref				
	Positive (unadjusted)	2.609	1.145	1.104	6.165	**0.029**
	Positive (adjusted^*^)	2.607	1.165	1.086	6.258	**0.032**
**PSA vs STI**	Negative	Ref				
	Positive (unadjusted)	1.250	0.502	0.569	2.747	0.579
	Positive (adjusted^#^)	1.074	0.445	0.476	2.419	0.864

*Adjusted for age, current contraceptive use, condom use and STIs.

#Adjusted for age, current contraceptive use, condom use and BV. Values shown in bold were those that were significant p < 0.05.

### Impact of PSA on Innate Factors in the Female Genital Tract

First, we determined the cytokine expression profiles in women with and without PSA. Unsupervised hierarchical clustering of cytokines identified no overt differences of cytokine expression profiles in women with or without PSA in their genital fluid (**Supplementary Figure 1A**). Principal component analysis (PCA) confirmed this finding, with no notable differences in principal component distribution of cytokines observed in women with detectable PSA versus those without PSA (**Supplementary Figure 1B**).

**Figure 1:**
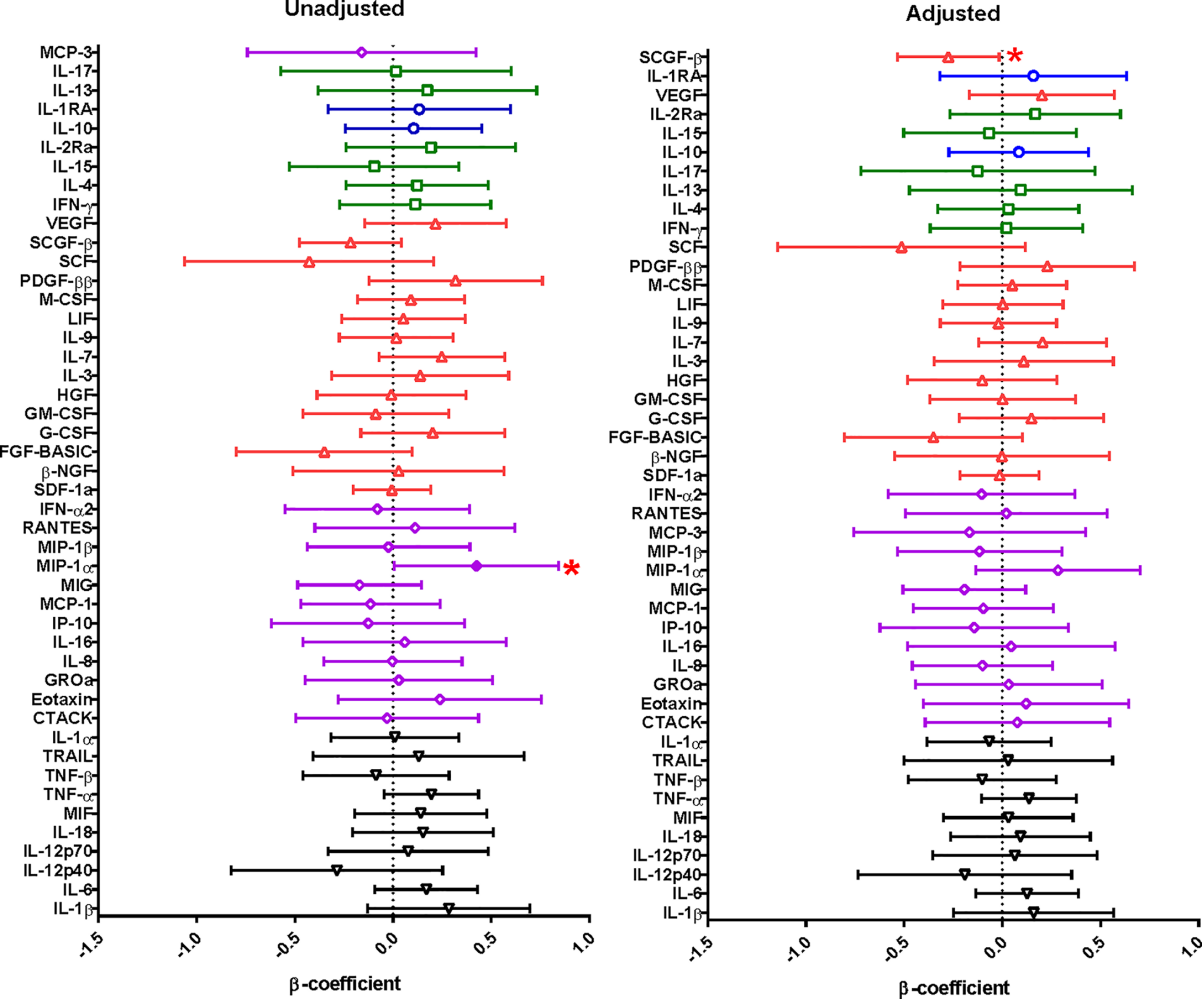
Linear regression model was used to evaluate the relationship between cytokine concentrations in menstrual cup supernatants and PSA from 248 HIV uninfected women. The cytokine concentrations were log-transformed and the cytokine concentrations were compared to PSA (whether positive or negative). The error bars indicate 95% confidence intervals. A significant association is shown by a shade circle and red asterisk (p<0.05). Unadjusted is for the univariate analysis and adjusted is for the multivariate analysis. Cytokine functions; pro-inflammatory – black inverted pyramid, chemokines – purple diamond, growth factors – red triangle, adaptive - green squares and anti-inflammatory cytokines – blue circles.

We then assessed the extent to which PSA impacted on menstrual cup supernatant cytokine milieu (**Figure 1**, **Supplementary Table 1**). In a univariate analyses, the concentrations of soluble factors MIP-1α (p=0.047) were higher in women with detectable PSA compared to women with no detectable PSA, but did not hold after adjusting for confounders such as age, condom use, STIs, BV and current contraceptive use. The concentrations of SCGF-β (p=0.038) were significantly decreased in menstrual cup supernatant of women with detectable PSA compared to without detectable PSA.

## Discussion

This study observed high levels of discordance between participant’s self-report of consistent condom use and PSA positivity. This is not particularly surprising especially in large observational research studies on reproductive and sexual health. Two-thirds of women who reported condom use with their partners to protect themselves from STIs tested positive for PSA. A positive PSA ELISA in women who reported 100% condom use likely indicates biased self-reporting of condom use or incorrect condom use (unprotected exposure to semen) by male partner during sexual act 48 hours prior sampling. Several studies have reported high rates (up to 38%) of breakage, leakage, slipping off, reuse, and the late application or early removal of condoms in young people (41, 42). Other than possible false positive results, inconsistencies between self-reported condom use and PSA positivity in menstrual cup supernatants may also be due to participants perceiving some topics as sensitive or the perceived fear of being non-compliant with barrier method use recommended during counselling sessions with study staff. Furthermore, use of hormonal contraception may contribute to inconsistencies between self-reported condom use and PSA positivity, as contraceptive users are less likely to use a condom (43). There were low levels of contraception use among the women in this study and this may be due to the fact that these women were more likely to be married, have a stable partner, or were trying to conceive.

Semen has been shown to serve as a medium for the transmission of bacterial communities between unprotected sexual partners (17, 44), resulting in changes in the vaginal microbial communities. Our study found that PSA positivity was associated with BV prevalence. Others have shown a microbial shift after unprotected sexual intercourse, resulting in a decreased abundance of *Lactobacillus* spp. and overgrowth of anaerobic BV-associated bacteria such as *Gardnerella vaginalis, Prevotella bivia, Atopobium vaginae* (17, 45, 46). In addition, a study among sex workers from three different African countries showed a significant association between BV, sex work and recent semen exposure (46). It is plausible to assume that the microbial changes brought by the presence of semen exposure are short lived and may be due to the alkaline pH found in semen. *Lactobacillus* spp. thrive in acidic environment with high glycogen content while they struggle in environments with pH greater than 4.5 (37, 47). In contrast, BV-associated bacterial species such as *Gardnerella, Prevotella, Atopobium* dominate in high pH environments (48–50). Despite this strong link between recent semen exposure and women with intermediate flora, no differences in prevalence of STIs (including *C. trachomatis, N. gonorrhoea, and T. vaginalis*) were found.

Previous studies have demonstrated a semen induced inflammatory response by endometrial epithelial cells *in vitro* (9, 51, 52). In agreement with previous studies, the current study found that menstrual cup supernatants of women detectable PSA increased expression of the MIP-1α (3) and a decreased expression of SCGF-β compared to those with no detectable PSA. Chemokine MIP-1α play a critical role during inflammation (5) and is primarily associated with cell adhesion and migration (53). Hematopoietic growth factor SCGF-β play an important role in restoring the barrier function and can support growth of primitive hematopoietic cells in the FGT (54). Taken together, an increased level of MIP-1α and lower concentrations of SCGF-β suggest that recent exposure to semen may lead to increased genital inflammation and weakened epithelial barrier in women with detectable PSA. The associations between cytokines and recent semen exposure should be interpreted conservatively as none of these associations were significant after adjusting for multiple comparisons and sample sizes for these analyses were relatively small. In addition, seminal fluids might dilute vaginal secretions and lead to lower concentrations of some cytokines in secretions.

This study had several limitations. Firstly, the study did not include Y chromosome data, which is indicative of unprotected sexual act with 15 days, so any interpretation of unprotected intercourse is limited to the past 48 hours. Secondly, there was a lack of information from the participants on self-reported timing and use of condoms in their most recent sex act. Thirdly, the present study could not evaluate the relationship between the presence of recent semen exposure, and incident and recurrent STIs and BV. This was attributed to small samples size of those who cleared BV/STI and had recurrence. Several studies have reported BV and/or STIs recurrence even after successful treatment (55, 56) and this recurrence has been attributed to biofilm (produced by microbes such as *G. vaginalis*) or reinfection from “BV/STI boyfriends”, an untreated sexual partner with BV and/or STIs (57). Lastly, the impact of recent semen exposure on FGT cytokines was assessed cross-sectionally instead of longitudinally, where analytes are investigated in the same women prior and post coitus. Furthermore, cytokine levels are higher in mucosal secretions from younger women compared to older women, yet this study did not age-match participants for subsequent cytokine analyses. Likewise, this study did not evaluate the impact of pH on cytokine milieu and BV incidence due to lack of data. Previous studies have demonstrated that seminal pH increased vaginal pH (58), altered vaginal microbiota with impairment in lactic acid producing lactobacilli colonization (15), and heightened genital inflammation (10). Lastly, we were unable to investigate the impact of other potential co-factors, including human papillomavirus, herpes simplex virus, hormonal contraceptives, menstrual cycles, hormonal status, other vaginal disorders (e.g., aerobic vaginitis), although these may have different biological effects.

## Conclusion

This study found a high level of discordance between self-report of condom use and recent semen exposure. We also found that recent semen exposure has a potential to alter the inflammatory response of the FGT and BV prevalence. Studies should measure PSA as an objective biomarker of unprotected sex and include it as a factor that needs to be adjusted for in the analysis to reduce the biases inherent to self-reporting of condom use and confounding effect of semen in participants of HIV prevention trials.

## Data Availability Statement

The original contributions presented in the study are included in the article/**Supplementary Material**. Further inquiries can be directed to the corresponding author.

## Ethics Statement

The studies involving human participants were reviewed and approved by Biomedical Research Ethics Committee of the University of KwaZulu Natal (BE316/17). The patients/participants provided their written informed consent to participate in this study.

## Author Contributions

KM and SN conceived and designed the study and KM, GM, AMt and SN performed the laboratory experiments. KM, GM, AMt, FO and SN analysed and interpreted the data while KM, GM, AMt, FO, LL, NG, AR, AMi, SA, QA, CB and SN have contributed to the interpretation and discussion of the results, and writing of the manuscript. All authors contributed to the article and approved the submitted version.

## Funding

This study was conducted as part of the DST-NRF Centre of Excellence (CoE) in HIV Prevention, which is supported by the Department of Science and Innovation and the National Research Foundation (grant 96354). The CAPRISA 083 parent study was funded by a United States – South African Program for Collaborative Biomedical Research grant through the South African Medical Research Council and the National Institute of Health (AI116759). KM was funded by DST-NRF CoE in HIV Prevention (grant 96354). SN was supported by Columbia University-Southern African Fogarty AITRP Programme (grant# D43TW00231), National Research Fund Thuthuka Research Grant (grant# TTK160510164586), and Poliomyelitis Research Foundation Research Grant (grant# 16/17).

## Conflict of Interest

The authors declare that the research was conducted in the absence of any commercial or financial relationships that could be construed as a potential conflict of interest.
