# Supplementary material for: Recent Semen Exposure Impacts the Cytokine Response and Bacterial Vaginosis in Women
Source: Front Immunol. 2021 Jun 9;12:695201. doi: 10.3389/fimmu.2021.695201 (PMC8221111; doi:10.3389/fimmu.2021.695201)
Supplement: Supplementary file 1 [file DataSheet_1.docx]

******

Supplementary Figure 1: Cytokine concentrations in women with PSA versus those without PSA. (A): Hierarchical clustering depicting cytokine expression profiles. Red and blue colours represent the standardized cytokine concentration values above and below zero, respectively. Yellow bars indicate women who tested positive for PSA while green indicate those without detectable PSA. The vertical axes represent the individual cytokines (right) and clusters (left); while horizontal axes represent participant identities. Cytokine values were scaled and centred for dendogram plotting. (B): Principal component analysis of cytokines in women with detectable PSA (red dots; n=43) compared to those with no PSA detectable (blue dots; n=205).
