# Supplementary material for: Recent Semen Exposure Impacts the Cytokine Response and Bacterial Vaginosis in Women
Source: Front Immunol. 2021 Jun 9;12:695201. doi: 10.3389/fimmu.2021.695201 (PMC8221111; doi:10.3389/fimmu.2021.695201)
Supplement: Supplementary file 2 [file Table_1.docx]

Supplementary Table 1: Effect of semen on cytokine/chemokine profiles in female genital tract secretions

| **Functional groups** | **Cytokines** | **PSA positive** | | **PSA negative** | | | **Univariate** | | ^#^**Multivariate** | |
| --- | --- | --- | --- | --- | --- | --- | --- | --- | --- | --- |
|  |  | **Median (pg/ml)** | **IQR** | **Median (pg/ml)** | | **IQR** | **β coefficient (SE)** | **P value** | **βcoefficient (SE)** | **P value** |
|  |  |  |  |  |  |  |  |  |  |  |
| **Pro-Inflammatory** | IL-1α | 3.964 | 3.345 - 4.357 | 3.898 | 3.354 - 4.422 | | 0.008 (0.165) | 0.961 | -0.067 (0.161) | 0.678 |
|  | IL-1β | 3.823 | 3.065 - 4.171 | 3.471 | 2.868 - 3.990 | | 0.284 (0.210) | 0.177 | 0.160 (0.207) | 0.441 |
|  | IL-6 | 2.809 | 2.414 - 3.264 | 2.634 | 2.182 - 3.057 | | 0.169 (0.133) | 0.204 | 0.126 (0.133) | 0.343 |
|  | IL-12p40 | 0.705 | 0.705 - 3.786 | 3.318 | 0.705 - 3.914 | | -0.285 (0.273) | 0.296 | -0.191 (0.276) | 0.490 |
|  | IL-12p70 | 3.230 | 2.750 - 3.557 | 3.124 | 2.705 - 3.487 | | 0.077 (0.207) | 0.710 | 0.064 (0.212) | 0.763 |
|  | IL-18 | 3.968 | 3.296 - 4.640 | 3.932 | 3.165 - 4.386 | | 0.153 (0.182) | 0.401 | 0.093 (0.180) | 0.608 |
|  | MIF | 4.844 | 4.184 - 5.285 | 4.825 | 3.893 - 5.228 | | 0.140 (0.171) | 0.413 | 0.032 (0.167) | 0.851 |
|  | TNF-α | 3.254 | 2.798 - 3.441 | 2.999 | 2.752 - 3.371 | | 0.195 (0.122) | 0.111 | 0.136 (0.122) | 0.266 |
|  | TNF-β | 2.205 | 1.597 - 2.567 | 2.101 | 1.624 - 2.641 | | -0.087 (0.189) | 0.644 | -0.101 (0.191) | 0.596 |
|  | TRAIL | 2.472 | -0.367 - 3.149 | 2.416 | -0.367 - 3.243 | | 0.130 (0.272) | 0.634 | 0.030 (0.270) | 0.912 |
| **Chemokines** | CTACK | 3.451 | 2.870 - 3.632 | 3.467 | 3.015 - 3.663 | | -0.030 (0.236) | 0.897 | 0.077 (0.239) | 0.747 |
|  | EOTAXIN | 2.640 | -0.352 - 2.898 | 2.485 | -0.352 - 2.976 | | 0.238 (0.263) | 0.367 | 0.121 (0.266) | 0.650 |
|  | GRO-a | 4.658 | 3.792 - 5.065 | 4.685 | 3.910 - 5.331 | | 0.029 (0.243) | 0.905 | 0.033 (0.241) | 0.891 |
|  | IL-8 | 4.466 | 4.103 - 4.854 | 4.516 | 4.051 - 5.017 | | -0.004 (0.181) | 0.981 | -0.101 (0.182) | 0.579 |
|  | IL-16 | 3.223 | 0.158 - 3.760 | 3.050 | 0.158 - 3.713 | | 0.059 (0.263) | 0.823 | 0.045 (0.268) | 0.867 |
|  | IP-10 | 4.177 | 3.440 - 4.607 | 4.329 | 3.408 - 4.985 | | -0.127 (0.249) | 0.610 | -0.143 (0.244) | 0.558 |
|  | MCP-1 | 3.038 | 2.809 - 3.235 | 3.143 | 2.916 - 3.332 | | -0.115 (0.180) | 0.524 | -0.096 (0.181) | 0.594 |
|  | MCP-3 | -0.629 | -0.629 - 2.892 | 1.262 | -0.629 - 2.926 | | -0.160 (0.296) | 0.589 | -0.166 (0.300) | 0.579 |
|  | MIG | 4.270 | 3.826 - 4.660 | 4.419 | 3.823 - 4.980 | | -0.171 (0.160) | 0.285 | -0.194 (0.159) | 0.225 |
|  | MIP-1α | 1.682 | 1.436 - 1.911 | 1.599 | 1.217 - 1.970 | | 0.424 (0.213) | **0.047** | 0.284 (0.212) | 0.181 |
|  | MIP-1β | 2.204 | 1.851 - 2.593 | 2.284 | 1.774 - 2.646 | | -0.023 (0.210) | 0.912 | -0.116 (0.212) | 0.585 |
|  | RANTES | 1.897 | -0.523 - 2.395 | 1.795 | -0.523 - 2.415 | | 0.111 (0.259) | 0.667 | 0.021 (0.261) | 0.936 |
|  | IFN-α2 | 2.634 | 2.163 - 2.857 | 2.616 | 2.035 - 2.945 | | -0.080 (0.239) | 0.738 | -0.106 (0.242) | 0.662 |
| **Growth Factors** | β-NGF | 1.794 | 1.354 - 1.997 | 1.661 | 1.156 - 2.125 | | 0.028 (0.272) | 0.917 | -0.002 (0.278) | 0.995 |
|  | FGF-Basic | 2.656 | -0.171 - 2.840 | 2.863 | 2.165 - 3.037 | | -0.349 (0.227) | 0.126 | -0.350 (0.230) | 0.129 |
|  | G-CSF | 4.729 | 4.246 - 5.005 | 4.471 | 3.798 - 5.010 | | 0.201 (0.186) | 0.280 | 0.149 (0.187) | 0.426 |
|  | GM-CSF | 3.093 | 2.719 - 3.198 | 3.186 | 2.891 - 3.352 | | -0.088 (0.189) | 0.639 | 0.002 (0.188) | 0.991 |
|  | HGF | 4.162 | 3.654 - 4.524 | 4.072 | 3.561 - 4.608 | | -0.009 (0.193) | 0.964 | -0.102 (0.193) | 0.599 |
|  | IL-3 | 3.475 | 3.032 - 3.767 | 3.507 | 3.053 - 4.204 | | 0.138 (0.229) | 0.546 | 0.109 (0.232) | 0.639 |
|  | IL-7 | 2.201 | 1.951 - 2.530 | 2.097 | 1.769 - 2.304 | | 0.248 (0.162) | 0.128 | 0.205 (0.165) | 0.214 |
|  | IL-9 | 2.317 | 2.050 - 2.546 | 2.338 | 2.067 - 2.555 | | 0.016 (0.147) | 0.911 | -0.020 (0.150) | 0.894 |
|  | LIF | 2.979 | 2.401 - 3.334 | 2.861 | 2.477 - 3.276 | | 0.052 (0.160) | 0.745 | 0.003 (0.156) | 0.986 |
|  | M-CSF | 3.767 | 3.452 - 4.240 | 3.860 | 3.518 - 4.105 | | 0.090 (0.138) | 0.516 | 0.051 (0.141) | 0.715 |
|  | PDGF-ββ | 2.956 | 2.514 - 3.316 | 2.763 | 2.268 - 3.182 | | 0.318 (0.224) | 0.158 | 0.229 (0.226) | 0.311 |
|  | SCF | -0.757 | -0.757-(3.010) | 2.315 | -0.757 - 3.191 | | -0.427 (0.322) | 0.186 | -0.513 (0.320) | 0.111 |
|  | SCGF-β | 2.073 | 2.073 - 2.073 | 2.073 | 2.073 - 2.073 | | -0.217 (0.132) | 0.101 | -0.275 (0.131) | **0.038** |
|  | SDF-1α | 3.840 | 3.537 - 4.085 | 3.958 | 3.551 - 4.159 | | -0.006 (0.100) | 0.949 | -0.013 (0.102) | 0.896 |
|  | VEGF | 4.313 | 3.763 - 4.672 | 4.183 | 3.734 - 4.584 | | 0.216 (0.183) | 0.239 | 0.202 (0.187) | 0.282 |
| **Adaptive** | IFN-γ | 3.227 | 2.947 - 3.572 | 3.135 | 2.761 - 3.507 | | 0.113 (0.195) | 0.561 | 0.022 (0.197) | 0.912 |
|  | IL-2*†* | -2.301 | -2.301 - (-2.301) | -2.301 | -2.301 - (2.301) | | - | - | - | - |
|  | IL-4 | 1.510 | 1.221 - 1.703 | 1.483 | 1.182 - 1.769 | | 0.122 (0.183) | 0.506 | 0.031 (0.182) | 0.863 |
|  | IL-5*†* | 1.035 | -0.745 - (2.044) | 1.306 | -0.745 - 1.815 | | - | - | - | - |
|  | IL-13 | 1.631 | 1.068 - 1.989 | 1.662 | 1.073 - 1.913 | | 0.174 (0.283) | 0.539 | 0.095 (0.288) | 0.743 |
|  | IL-15 | -0.561 | -0.561-(1.631) | -0.561 | -0.561 - 1.833 | | --0.096 (0.216) | 0.663 | -0.067 (0.221) | 0.760 |
|  | IL-17 | 2.392 | 2.120 - 2.713 | 2.551 | 2.042 - 2.945 | | 0.015 (0.298) | 0.959 | -0.125 (0.303) | 0.681 |
|  | IL-2RA | 2.752 | 2.062 -3.081 | 2.711 | 2.150 - 3.182 | | 0.192 (0.219) | 0.382 | 0.168 (0.221) | 0.447 |
| **Anti-Inflammatory** | IL-10 | 2.859 | 2.450 - 3.154 | 2.853 | 2.444 - 3.128 | | 0.105 (0.176) | 0.552 | 0.084 (0.181) | 0.643 |
|  | IL-1RA | 8.425 | 6.115 - 8.425 | 8.425 | 6.248 - 8.425 | | 0.133 (0.236) | 0.574 | 0.158 (0.241) | 0.512 |
